# Mainstream genetic testing for women with ovarian cancer provides a solid basis for patients to make a well-informed decision about genetic testing

**DOI:** 10.1186/s13053-022-00238-w

**Published:** 2022-09-08

**Authors:** Kyra Bokkers, Eveline M. A. Bleiker, Jacob P. Hoogendam, Mary E. Velthuizen, Henk W. R. Schreuder, Cornelis G. Gerestein, Joost G. Lange, Jacqueline A. Louwers, Marco J. Koudijs, Margreet G. E. M. Ausems, Ronald P. Zweemer

**Affiliations:** 1 Department of Genetics, Division Laboratories, Pharmacy and Biomedical Genetics, University Medical Center Utrecht, Heidelberglaan 100, 3584 CX Utrecht, the Netherlands; 2 Division of Psychosocial Research and Epidemiology, The Netherlands Cancer Institute, Plesmanlaan 121, 1066 CX Amsterdam, The Netherlands; 3 Family Cancer Clinic, The Netherlands Cancer Institute, Plesmanlaan 121, 1066 CX Amsterdam, The Netherlands; 4 Department of Clinical Genetics, Leiden University Medical Center, Albinusdreef 2, 2333 ZA Leiden, The Netherlands; 5 Department of Gynecological Oncology, University Medical Center Utrecht, Heidelberglaan 100, PO Box 85500, 3584 CX Utrecht, The Netherlands; 6 Department of Gynecology, Meander Medical Center, Maatweg 3, 3813 TZ Amersfoort, The Netherlands; 7 Department of Gynecology, St. Antonius Hospital, Koekoekslaan 1, 3435 CM Nieuwegein, the Netherlands; 8 Department of Gynecology, Diakonessenhuis, Bosboomstraat 1, 3582 KE Utrecht, The Netherlands

**Keywords:** Epithelial ovarian cancer, Mainstream genetic testing, Patients’ perspectives, Genetic counseling, Turnaround times, Psychosocial outcomes, Knowledge, Satisfaction

## Abstract

**Background:**

There is a growing need for genetic testing of women with epithelial ovarian cancer. Mainstream genetic testing provides an alternative care pathway in which non-genetic healthcare professionals offer pre-test counseling themselves. We aimed to explore the impact of mainstream genetic testing on patients’ experiences, turnaround times and adherence of non-genetic healthcare professionals to the mainstream genetic testing protocol.

**Methods:**

Patients receiving pre-test counseling at the gynecology departments between April 2018 and April 2020 were eligible to participate in our intervention group. Patients receiving pre-test counseling at the genetics department between January 2017 and April 2020 were eligible to participate in our control group. We evaluated patients’ experiences with questionnaires, consisting of questions regarding knowledge, satisfaction and psychosocial outcomes. Patients in the intervention group were sent two questionnaires: one after pre-test counseling and one after receiving their DNA test result. Patients in our control group were sent one questionnaire after receiving their test result. In addition, we collected data regarding turnaround times and adherence of non-genetic healthcare professionals to the mainstream genetic testing protocol.

**Results:**

Participation was 79% in our intervention group (105 out of 133 patients) and 60% in our control group (91 out of 152 patients). Knowledge regarding genetics, decisional conflict, depression, anxiety, and distress were comparable in the two groups. In the intervention group, the risk of breast cancer in patients carrying a pathogenic germline variant was discussed less often (49% versus 74% in control group, *p* ≤ 0.05), and the mean score of regret about the decision to have genetic testing was higher than in the control group (mean 12.9 in the intervention group versus 9.7 in the control group, *p* ≤ 0.05), although below the clinically relevant threshold of 25. A consent form for the DNA test and a checklist to assess family history were present for ≥ 95% of patients in the intervention group.

**Conclusion:**

Mainstream genetic testing is an acceptable approach to meet the increase in genetic testing among women with epithelial ovarian cancer.

**Supplementary Information:**

The online version contains supplementary material available at 10.1186/s13053-022-00238-w.

## Introduction

Genetic testing for patients with ovarian cancer has increased over the years, due to expanding eligibility criteria and individualized treatment options that are dependent on DNA test results. All patients with epithelial ovarian cancer (EOC) are eligible for genetic testing [1–3]. Patients with platinum-sensitive EOC are sensitive to treatment with PARP inhibitors, with an increased response when a pathogenic variant in a *BRCA* gene is present [2, 4].

With mainstream genetic testing, non-genetic healthcare professionals (HCPs) perform pre-test counseling and order germline genetic testing for their patients [5, 6]. Additional counseling by a genetic counselor or clinical geneticist is only required in case of a pathogenic variant or variant of unknown significance in a cancer predisposition gene. The importance of genetic testing for patients with EOC and low referral rates to genetics departments in the past have led to the rise of mainstream genetic testing initiatives around the world [7, 8].

We have previously implemented a mainstream genetic testing pathway in four hospitals in the Netherlands, and we have shown that gynecologic oncologists, gynecologists with a subspecialty training in oncology, and nurse specialists feel capable of performing pre-test counseling and ordering genetic testing themselves and are motivated to do so [9]. Earlier research has shown that patients with EOC appreciate being offered a DNA test shortly after diagnosis [10–13], and their distress and cancer worry do not increase following genetic counseling [11, 14].

However, with mainstream genetic testing, non-genetic HCPs need to incorporate genetic testing into their routine practice. The time spent on pre-test counseling may be considerably shorter compared to the duration of the pre-test counseling performed by clinical geneticists or genetic counselors. In addition, with mainstream genetic testing there is no wait time for patients to receive pre-test counseling. This is beneficial for possible treatment options, but also eliminates a time period for patients to consider genetic testing before their first pre-test counseling. This may result in more distress or decisional conflict or regret in patients.

Because of these differences in the clinical setting, it is impossible for non-genetic HCPs to provide the same pre-test counseling as provided by a clinical geneticist or genetic counselor. These differences are acceptable as long as patients are able to make a well-informed decision regarding genetic testing without experiencing excessive distress or regret. In addition, non-genetic HCPs need to incorporate an informed consent procedure and identify patients who might benefit from additional counseling at a genetics department, for example for genetic testing for Lynch syndrome.

Many studies have shown high acceptability of mainstream genetic testing approaches among EOC patients [5, 6, 15–21]. So far, these outcomes have only been evaluated sporadically with a control group receiving pre-test genetic counseling at a genetics department [16, 18–20]. Two of these studies included both patients with breast and ovarian cancer, and post-test counseling was always performed by a genetic counselor or clinical geneticist [16, 19]. Another study predominantly considered patient satisfaction [18].

In this study, we will assess the impact of mainstream genetic testing on patient care in comparison to genetic counseling and testing performed by a clinical geneticist or genetic counselor. The impact on patient care is evaluated based on psychosocial outcomes, knowledge and satisfaction of patients, turnaround times, and the adherence of non-genetic HCPs to the mainstream genetic testing protocol.

## Material and methods

### Mainstream genetic testing pathway

We previously described the development and workflow of our mainstream genetic testing pathway [9]. We implemented this pathway in the four hospitals in our region where patients are diagnosed and treated for EOC. In April and August 2018, we started in the two hospitals with the highest numbers of newly diagnosed patients with EOC. In March and July 2019, we implemented our pathway in the other two hospitals. After completion of a training module, non-genetic HCPs could perform pre-test genetic counseling and order genetic testing for all patients eligible for genetic testing according to national guidelines (i.e., EOC, including fallopian tube and extra ovarian carcinomas), including patients who were diagnosed in the past and had not yet received genetic testing [1]. These non-genetic HCPs included gynecologic oncologists, gynecologists with a subspecialty training in oncology, and nurse specialists. If indicated by the patient or non-genetic HCP, patients could still be referred for pre-test counseling by a genetic HCP (e.g., when the patient had questions that the non-genetic HCP could not answer). Our gene panel first consisted of the genes: *BRCA1* and *BRCA2* [1]*.* During our study, this panel was complemented by the genes *BRIP1, RAD51C,* and *RAD51D*.

During pre-test counseling, non-genetic HCPs informed patients of the implications of genetic testing and handed out an information sheet with general information about genetic testing. For patients who accepted genetic testing, written informed consent was obtained and the DNA test ordered. In addition, non-genetic HCPs filled out a checklist to identify patients with a relevant personal or family history indicative for referral to a genetics department (e.g., meeting eligibility criteria for Lynch syndrome testing and/or preventive measures for family members).

The genetics department sent the test results to patients in a letter, which also included a general information sheet explaining this result. This letter was also sent to the HCP who had ordered the DNA test and to the general practitioner. An invitation for post-test counseling at the genetics department was added to this letter for all patients carrying a pathogenic variant or variant of unknown significance within five working days, or patients with a relevant personal or family history within 6–8 weeks.

### Standard genetic testing pathway

For patients referred to the genetics department, a clinical geneticist or genetic counselor performed pre-test counseling and acquired information regarding the family history, obtained written informed consent and ordered the DNA test. During our study period, patients could either be referred to the genetics department by non-genetic HCPs who were not trained in the mainstream genetic testing pathway (e.g., general practitioners or medical oncologists) or by trained non-genetic HCPs when there was an indication for such a referral. Test results were discussed with the patient in person, via telephone or videoconference. Subsequently, the test result and possible implications of this result for patient and family members were summarized in a letter to the patient. This letter was also sent to the general practitioner and the non-genetic HCP who referred the patient.

### Study design and participants

**Fig. 1:**
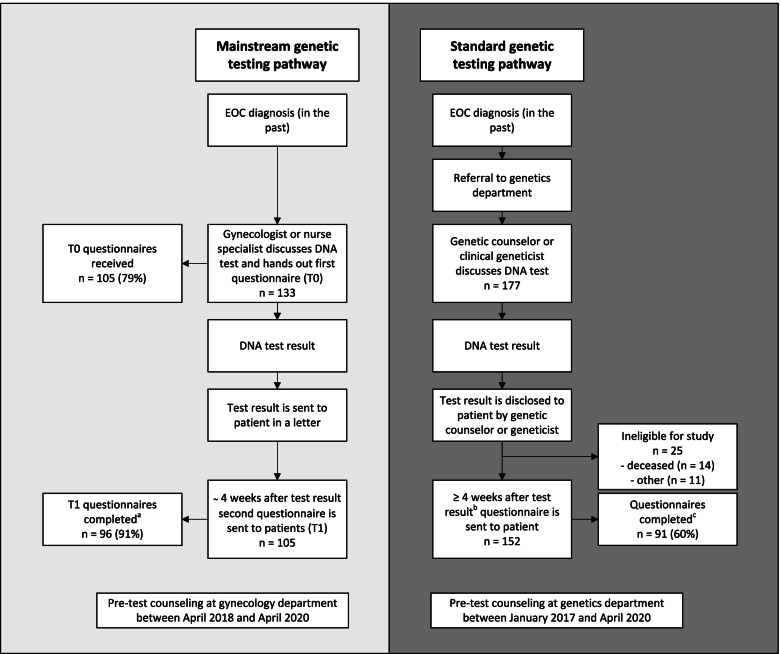
Study design and participation in questionnaire study. ^a^Two questionnaires were returned without being completed and with a comment that the patient had died. ^b^Patients in the control group received pre-test genetic counseling both before and during our study period (from January 2017 until April 2020). Patients who received genetic counseling during our study period received the questionnaire approximately four weeks after the test result was made available. Patients who received genetic counseling before our study period received the questionnaire between four weeks and one year after receiving the test result. ^c^Two patients were excluded after receiving the questionnaire, one because of a language barrier and one because the patient received counseling for breast cancer and the EOC was diagnosed after preventive surgery

All patients who received pre-test genetic counseling and testing in the mainstream genetic testing pathway were invited prospectively to participate in our questionnaire study between April 2018 and April 2020 (see Fig. 1). All patients who received pre-test counseling were eligible to participate in our intervention group, even if they declined genetic testing. They received information about the study, including a response sheet, directly after discussing the DNA test with their HCP (T0). We sent a reminder letter after two weeks to all patients for whom a DNA test was requested by a non-genetic HCP. The first questionnaire was sent to patients who accepted the invitation to participate in our study. Patients only received a second questionnaire if a DNA test was performed. This second questionnaire was sent to patients approximately four weeks after receiving their test result (T1).

For our control group, we retrospectively invited patients who had received pre-test genetic counseling and testing in the standard genetic testing pathway to participate in our questionnaire study at least four weeks after receiving the test result. We identified all patients with EOC who had received pre-test counseling at the genetics department between January 2017 and April 2020. We only invited patients to participate in our study when we could confirm vital status and current address. In addition, we excluded patients who previously declined to participate in research, had not completed their genetic counseling, or when a pathogenic variant in one of the ovarian cancer genes was already identified in a family member. We sent out a reminder letter after two weeks to non-responders.

We obtained data from medical records of patients who participated in our questionnaire study regarding: diagnosis, age at diagnosis, interval between receiving test result and completing the questionnaire, turnaround times, genes tested, and test results. The consent forms for diagnostic germline genetic testing and checklists evaluating patients’ personal and family history were only evaluated for patients in the intervention group. For the evaluation of these consent forms, checklists and, in addition, turnaround times, our intervention group consisted of all patients who received mainstream genetic testing, and not only the patients who participated in our questionnaire study.

### Questionnaires

The questionnaires consisted of nine elements: (1) sociodemographics, (2) treatment history, (3) distress, (4) anxiety and depression, (5) knowledge, (6) discussed topics during pre-test counseling, (7) satisfaction with pre-test counseling, (8) satisfaction with receiving the test result, and (9) satisfaction with the decision to accept or decline genetic testing. Table 1 shows which elements were present in the different questionnaires for the intervention and control group.

**Table 1:** Overview of topics in questionnaires

		**Intervention group**		**Control group**
		***T0 questionnaire***	***T1 questionnaire***	
*Elements*	*Tool*			
Sociodemographics		x		x
Treatment history		x		x
Distress	- DT	x	x	x
Anxiety and depression	- HADS	x	x	x
Knowledge		x	x	x
Discussed topics during pre-test counseling		x		x
Satisfaction with pre-test counseling		x		x
Satisfaction with receiving the test result			x	x
Satisfaction with the decision to accept or decline genetic testing	- DCS	x	x	x
	- DRS		x	x

*DT* Distress Thermometer*, HADS* Hospital Anxiety and Depression Scale*, DCS* Decisional Conflict Scale*, DRS* Decision Regret Scale

### Outcome measures

#### Psychosocial outcomes

Psychosocial outcomes consisted of (1) anxiety and depression, (2) distress, (3) decisional conflict, and (4) decision regret.

Anxiety and depression were measured using the Hospital Anxiety and Depression Scale (HADS) [22, 23]. The HADS is a validated questionnaire consisting of 14 items with a four-point Likert scale: seven questions for anxiety (HADS-A) and seven questions for depression (HADS-D). Scores for both subscales range between zero and 21. Scores on a subscale ≥ 11 indicate clinically significant levels of anxiety or depression [24].

Distress was measured using the one-item Distress Thermometer (DT) [25]. The DT has a scale from 0 to 10, with 0 indicating ‘no distress’ and 10 indicating ‘extreme distress’. A score of ≥ 4 indicates moderate to severe distress [25].

Decisional conflict was measured with the decisional conflict scale [26, 27]. This questionnaire consists of 16 items with a five-point Likert scale for each question. A total score and five subscores can be determined, all ranging from 0 to 100, with 0 indicating no decisional conflict and 100 indicating maximal decisional conflict. The question: ‘*I expect to stick with my decision’* was left out of the T1 questionnaire for the intervention group and questionnaire for the control group because these questionnaires were sent after the DNA test had already been performed and therefore this question did not apply at that time.

The level of decision regret was measured with the decision regret scale [28]. This questionnaire consists of five items with a 5-point Likert scale. Scores range between 0 and 100, with 0 indicating no regret and 100 indicating maximal regret.

#### Knowledge and discussed topics

Knowledge was measured with five statements adapted from Claes et al. that can be answered with ‘true’, ‘false’ or ‘don’t know’ [29].

Discussed topics consisted of (1) consequences for patients’ treatment, (2) possible implications for family members, and (3) the associated higher risk of developing breast cancer if a pathogenic variant in a *BRCA* gene is found. Patients were able to select one or more of these three options and were asked to select the topic that was most important to them.

#### Satisfaction

The patients’ satisfaction with pre-test counseling and how they received the test result were measured using self-developed questions, derived from the questionnaires used in the Mainstreaming Cancer Genetics (MCG) program and developed for the TIME trial, which evaluated breast cancer patients’ experiences with rapid genetic testing and counseling [6, 30].

#### Turnaround times

For both groups, we evaluated the time between diagnosis, pre-test counseling, and communicating the test result to the patient. For patients in the control group, we also included the time of referral. For patients in the intervention group, we also included the time of additional post-test counseling at the genetics department, if applicable.

We used the date of the histology report as the time of diagnosis. If a histology report was lacking, the date of the cytology report was used. For patients in the intervention group, we used the date that the letter with the test result was sent to the patient as the time that the test result was communicated to the patient. For patients in the control group, we used the date that the test result was first communicated to the patient, which was foremost the date of a telephone consultation.

If the month and/or day of the date were missing, June and/or the 15^th^ were added in order to be able to calculate the turnaround times.

#### Adherence to the mainstream genetic testing protocol

We assessed whether written informed consent was obtained for diagnostic germline genetic testing based on the presence of a consent form in the patient file. In addition, we assessed whether non-genetic HCPs evaluated whether the patient required additional post-test counseling at the genetics department based on patient or family history. We determined this based on the presence of the checklist in the patient file. We also assessed whether or not patients were actually referred to the genetics department if indicated by this checklist.

### Statistical analyses

We calculated mean and standard deviation or median and range for continuous variables and frequencies and percentages for categorical variables. Groups were compared using univariate analysis with logistic regression or a chi-square test for categorical variables and linear regression for continuous variables. We performed multivariate analyses on the decisional conflict scale, the decision regret scale, the HADS and DT. We adjusted for the possible confounders, based on literature and expert opinion: having a pathogenic variant or variant of unknown significance, having one or more children, educational level, having a personal history of another type of cancer in addition to the EOC diagnosis, the interval between receiving the DNA test result and completing the questionnaire, and being offered genetic testing ≤ 6 months after diagnosis. We imputed (five times) the missing data (< 6%) of these outcomes and possible confounders. For the calculation of the turnaround times, we excluded the extreme outliers. We defined extreme outliers as values that were either 3 times the interquartile range above the 3^rd^ quartile value or 3 times below the 1^st^ quartile. IBM SPSS statistics 26.0.0.1 was used to perform the statistical analyses.

## Results

### Participation and patient characteristics

During our study period, non-genetic HCPs requested a DNA test for 133 patients, of whom 105 (79%) participated in our study (intervention group). We received 105 T0 questionnaires and 96 T1 questionnaires. We identified 177 patients with EOC who had received pre-test counseling at the genetics department between January 2017 and April 2020. In total, 152 patients were eligible to participate in our questionnaire study, and 91 of these patients (60%) completed the questionnaire (control group). See also Fig. 1.

For both groups, we did not receive any questionnaires from patients who declined genetic testing. Because the control group was invited retrospectively, there was a longer period of time (*p* = 0.000) between receiving the test result and completing the questionnaire (mean 232 days, sd 14.6) compared to the intervention group (mean 57 days, sd 3.1).

The patient characteristics are shown in Table 2. The study group consisted mainly of patients with high-grade serous EOC. Most patients had one or more children, an intermediate educational level and a Dutch native background. In our intervention group, the mean age was higher and there were significantly more patients who had one or more children. There were no statistically significant differences between the responders and non-responders in the intervention group with regard to age at diagnosis, whether patients were newly diagnosed at time of pre-test counseling or not, histology, types of genes tested and test result (data not shown).

**Table 2:** Patient characteristics

	**Intervention group, *n* = 105**	**Control group, *n* = 91**	***P*-value**
Age at diagnosis, mean (sd)	67.4 (9.6)	63.0 (11.1)	0.003*
Newly diagnosed at time of being offered genetic testing^a^, n (%)	91 (86.7)	62 (68.1)	0.002*
Histology, n (%)	78 (74.3)	64 (70.3)	0.183
- Serous,	- 72	- 51	
- high grade	- 5	- 6	
- low grade	- 1	- 7	
- grade unknown	5 (4.8)	6 (6.6)	
- Endometrioid clear cell	5 (4.8)	4 (4.4)	
- Mucinous	8 (7.6)	7 (7.7)	
- Other/unknown	9 (8.6)	10 (11.0)	
DNA test results, n (%)			
- Normal	95 (90.5)	74 (81.3)	0.068
- Pathogenic variant or variant of unknown significance	10 (9.5)	17 (18.7)	
Children, n (%)			
- No	12 (11.4)	20 (22.0)	0.038*
- Yes	92 (87.6)	67 (73.6)	
- Unknown	1 (1.0)	4 (4.4)	
Educational level^b^, n (%)			
- Low	9 (8.6)	9 (9.9)	0.851
- Intermediate	71 (67.6)	58 (63.7)	
- High	24 (22.9)	23 (25.3)	
- Unknown	1 (1.0)	1 (1.1)	
Migrant status^c^, n (%)			
- Dutch Native	92 (87.6)	82 (90.1)	0.946
- Migrant	10 (9.5)	8 (8.8)	
- Western	- 8	- 6	
- Non-Western	- 2	- 2	
- Unknown	3 (2.9)	1 (1.1)	
Personal history of another type of cancer, n (%)	16 (15.7)	15 (17.2)	0.774

^a^ Newly diagnosed at time of being offered genetic testing was defined as receiving pre-test counseling ≤ 6 months after diagnosis for the intervention group and being referred to the genetics department ≤ 6 months after diagnosis for the control group

^b^ Educational level is subdivided into low, intermediate or high level as categorized by the Dutch Standard Classification of Education 2021 [31]. Low level education is no education, primary education or lower secondary education, intermediate level education is upper secondary education and high-level education is tertiary education

^**c**^ Migrant status is defined by Statistics Netherlands (CBS) as having at least one parent who was born abroad [32]. A distinction can be made between a Western migration background (country of origin in Europe (excluding Turkey), North America, and Oceania, or from Indonesia or Japan) and a non-Western migration background (country of origin in Africa, South America or Asia (excluding Indonesia and Japan) or from Turkey). If a person is born in the Netherlands, the migration background is determined by the mother’s country of birth. When the mother is born in the Netherlands as well, then the migration background is determined by the father’s country of birth

^*^p ≤ 0.05

### Psychosocial outcomes

Table 3 shows the decisional conflict, decision regret, anxiety, depression and distress for both groups. The univariate analyses did not show any significant differences in decisional conflict or decision regret between the two groups. When corrected for our confounders with multivariate analyses, decision regret was significantly higher in our intervention group. There were no significant differences for anxiety, depression, or distress between the two groups with univariate and multivariate analyses.

**Table 3:** Decisional conflict, decision regret, anxiety, depression and distress

			*univariate*		*multivariate*	
	Intervention group, *n* = 96	Control group, *n* = 91	mean diff or OR (95% CI)	*p*-value	mean diff or OR (95% CI)	*p-*value
**Decisional conflict scale, mean (sd)**						
- total score	19.7 (12.0)	19.3 (14.4)	0.4 (-3.5 – 4.3)	0.844	2.7 (-2.4 – 7.8)	0.294
- uncertainty subscore	17.5 (19.8)	20.0 (21.3)	-2.5 (-8.6 – 3.5)	0.408	3.5 (-4.7 – 11.8)	0.402
- informed subscore	20.2 (19.8)	19.1 (18.0)	1.1 (-3.7 – 5.9)	0.655	2.4 (-4.1 – 8.8)	0.472
- support subscore	19.5 (13.9)	19.4 (16.9)	0.1 (-4.4 – 4.7)	0.947	0.4 (-5.8 – 6.7)	0.889
- values clarity subscore	25.0 (16.2)	24.0 (17.4)	1.0 (-4.0 – 5.9)	0.702	2.9 (-3.8 – 9.6)	0.400
- effective decision subscore	16.8 (15.7)	15.1 (14.8)	1.8 (-2.7 – 6.2)	0.433	3.8 (-2.3 – 10.0)	0.215
**Decision regret scale, mean (sd)**						
- total score	12.9 (13.2)	9.7 (11.0)	3.2 (-0.4 – 6.7)	0.079	4.9 (-0.7 – 9.7)	0.047*
**HADS-Anxiety, mean (sd)**	5.7 (4.1)	5.3 (3.7)				
**Subgroups, n (%)**						
- ≤ 10	84 (87.5)	75 (82.4)	1.3 (0.6 – 3.1)	0.523	2.7 (0.8 – 9.2)	0.101
- ≥ 11	11 (11.5)	13 (14.3)				
- missing	1 (1.0)	3 (3.3)				
**HADS-Depression, mean (sd)**	4.6 (4.1)	3.6 (3.7)				
**Subgroups, n (%)**						
- ≤ 10	84 (87.5)	86 (94.5)	0.5 (0.2 – 1.7)	0.291	0.7 (0.1 – 3.7)	0.715
- ≥ 11	9 (9.4)	5 (5.5)				
- missing	3 (3.1)	0				
**Distress Thermometer, mean (sd)**	3.9 (2.5)	3.7 (2.5)				
**Subgroups, n (%)**						
- ≤ 3	46 (47.9)	49 (53.8)	0.8 (0.4 – 1.4)	0.418	0.6 (0.3 – 1.4)	0.270
- ≥ 4	50 (52.1)	42 (46.2)				
- missing	0	0				

Decisional conflict and decision regret are measured on a scale of 0 to 100, with a higher score indicating more decisional conflict or regret

For the continuous variables, i.e., decisional conflict scale and decision regret scale, the difference between the mean score for the control group and the intervention is shown (mean diff). For the dichotomous variables, i.e., HADS-Anxiety, HADS-Depression and Distress Thermometer, the odds ratio between the two groups is shown

*OR* Odds Ratio, *CI* Confidence interval

^*^p ≤ 0.05

### Knowledge and discussed topics

The average number of correct answers for the five knowledge statements was 3.0 (sd 1.6) in the intervention group, and 3.3 (sd 1.4) in the control group (*p* = 0.155). Considering the individual statements, patients in the intervention group scored significantly worse on the statement ‘A woman who has a sister with a pathogenic variant (gene alteration) in an ovarian cancer gene has a 50% chance (1 in 2) of having this gene alteration as well’ compared to the control group. Comparisons between the two groups for every individual statement are shown in Supplementary Table 1.

**Fig. 2:**
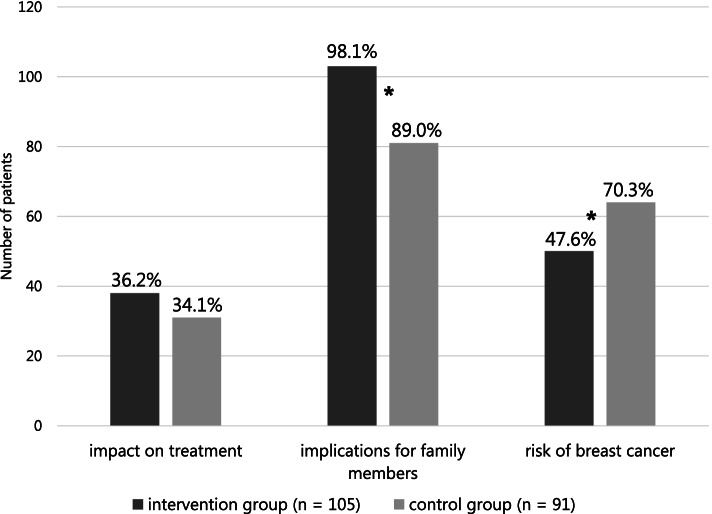
Discussed topics during pre-test counseling. The figure shows the percentage of patients who reported whether the following topics were discussed: (1) women with ovarian cancer and a pathogenic variant in an ovarian cancer gene can sometimes receive additional treatment if the ovarian cancer comes back later, (2) for family members it may be important to know if a woman with ovarian cancer has a pathogenic variant in an ovarian cancer gene, and (3) when a woman with ovarian cancer has a pathogenic variant in a *BRCA1* or *BRCA2* gene, she also has a higher chance of developing breast cancer. *p ≤ 0.05

The discussed topics during pre-test counseling are shown in Fig. 2. The possible impact of the DNA test result on the treatment were discussed with only a third of both groups, according to the patients. Patients in both groups reported that the possible implications for family members were most important to them: 72% of patients in the intervention group and 65% of patients in the control group.

### Patient satisfaction

Questions regarding satisfaction of patients with pre-test counseling and receiving their test result are shown in Tables 4 and 5. In the intervention group a significantly higher proportion of patients indicated that it did not matter to them how they received their test result. In addition, a significantly higher proportion of patients in this group answered that they were unsure whether or not they had enough time to weigh the advantages and disadvantages of a DNA test.

**Table 4:** Questions indicating satisfaction with pre-test counseling

	Options	Intervention group, *n* = 105	Control group, *n* = 91	*P*-value
Clarity of discussed information regarding the DNA test, n (%)	- (very) clear	98 (93.3)	89 (97.8)	0.156
	- unsure/not clear	7 (6.7)	2 (2.2)	
Received written information after discussing DNA test, n (%)	- yes	81 (77.1)	not asked^a^	N/A
	- no	22 (21.0)		
	- missing	2 (1.9)		
Clarity of received written information after discussing the DNA test, n (%)	- (very) clear	75 (92.6)	88 (96.7)	0.239
	- unsure/not clear	6 (7.4)	3 (3.3)	
There was enough time to weigh the advantages and disadvantages of a DNA test, n (%)	- yes	80 (76.2)	86 (94.5)	0.002*
	- no	4 (3.8)	1 (1.1)	
	- don’t know	21 (20.0)	4 (4.4)	
Feeling of having a choice whether or not to perform a DNA test, n (%)	- yes	97 (92.4)	79 (86.8)	0.082
	- no	3 (2.9)	9 (9.9)	
	- don’t know	5 (4.8)	2 (2.2)	
	- missing	0	1 (1.1)	
Satisfaction with being offered a DNA test, n (%)	- (very) satisfied	95 (90.5)	88 (96.7)	0.134
	- unsure/not satisfied	9 (8.6)	3 (3.3)	
	- missing	1 (1)	0	
Preferred moment to be offered a DNA test, n (%)	- directly after diagnosis	57 (54.3)	45 (49.5)	0.236
	- during treatment	3 (2.9)	2 (2.2)	
	- after completion of treatment	30 (28.6)	38 (41.8)	
	- in case of recurrence	4 (3.8)	4 (4.4)	
	- other	8 (7.6)	2 (2.2)	
	- missing	3 (2.9)	0	

*N/A* Not applicable

^*^p ≤ 0.05

^a^ For the control group it was assumed that all patients did receive written information after discussing the DNA test and therefore this was not asked in the questionnaire

**Table 5:** Questions indicating satisfaction with receiving test result

	Options	Intervention group, *n* = 96	Control group, *n* = 91	*P*-value
It was clear how the test result would be communicated, n (%)	- yes	85 (88.5)	86 (94.5)	0.057
	- no	9 (9.4)	2 (2.2)	
	- missing	2 (2.1)	3 (3.3)	
Clarity of written information about the test result, n (%)	- (very) clear	88 (91.7)	83 (91.2)	0.161
	- unsure/not clear	5 (5.2)	1 (1.1)	
	- missing	3 (3.1)	7 (7.7)	
Looking back information was missed to consider the DNA test, n (%)	- yes	4 (4.2)	4 (4.4)	0.949
	- no	88 (91.7)	84 (92.3)	
	- missing	4 (4.2)	3 (3.3)	
Number of days between pre-test counseling and communicating test result to patient, median (range)		36 (11 – 366)	55 (15 – 112)	0.055
Satisfied with number of days between pre-test counseling and receiving test result, n (%)	- (very) satisfied	78 (81.3)	71 (78.0)	0.467
	- unsure/not satisfied	14 (14.6)	17 (18.7)	
	- missing	4 (4.2)	3 (3.3)	
Ways of receiving test result, n (%)	- letter	N/A (all via a letter)	14 (15.4)	N/A
	- telephone		39 (42.9)	
	- consultation at genetics department		33 (36.3)	
	- other		1 (1.1)	
	- missing		4 (4.4)	
Satisfied with how test result was received, n (%)	- yes	59 (61.5)	75 (82.4)	0.002*
	- no	1 (1.0)	0	
	- no preference	34 (35.4)	13 (14.3)	
	- missing	2 (2.1)	3 (3.3)	

N/A Not applicable

^*^p ≤ 0.05

### Turnaround times

The turnaround times in the intervention group were significantly shorter than those in the control group, see Table 6.

**Table 6:** Turnaround times for the genetic testing pathways

Days between:	**Intervention group** ***n* = 133**	**Control group** ***n* = 91**	***p*-value**
Diagnosis and pre-test counseling, median (range)	45^a^ (-29^b^ – 260)	194^c^ (6 – 592)	0.000*
- diagnosis and referral	N/A	72^c^ (-3 – 575)	N/A
- referral and pre-test counseling	N/A	70 (-3 – 240)	N/A
pre-test counseling and communicating test result to patient, median (range)	35^d^ (11 – 72)	55 (15 – 112)	0.000*
sending letter with test result to patient and additional appointment at genetics department, median (range), *n* = 21	6 (0 – 58)	N/A	N/A
- normal result, *n* = 7	20 (6 – 42)		
- pathogenic variant or variant of unknown significance, *n* = 14	5.5 (0^e^ – 58^f^)		

*N/A* Not applicable. All turnaround times are presented in calendar days

^*^p ≤ 0.05

^a^ Based on 121 cases, 12 extreme outliers were excluded

^b^ One patient was invited for pre-test counseling because a relative of hers had received genetic counseling. Her referral followed after she already had pre-test counseling

^c^ Based on 78 cases, 13 extreme outliers were excluded

^d^ Based on 128 cases, 5 extreme outliers were excluded

^e^ For one patient the number of days between test result and additional appointment was 0 days, because the result was not sent in a letter, but the clinical geneticist visited the patient while she was admitted in the hospital

^f^ One patient postponed post-test counseling until she had completed her treatment

### Adherence to the protocol

The checklist to assess whether the patient had a relevant personal or family history for referral to a genetics department was present in the patient file for 126 out of 133 patients (94.7%). For 14 patients, there was a reason to refer the patient to the genetics department based on their checklist. Three of these patients (21.4%) had not been referred to the genetics department. The checklist of one of these patients was already assessed by the genetics department at time of the test result and they agreed that a referral was not necessary. For the other two patients, it was not clear why they were not referred. A signed consent form for diagnostic genetic testing was present in the electronic patient file of 130 patients (97.7%).

## Discussion

In this study, we evaluated the impact of mainstream genetic testing on genetic care of patients with EOC, based on patients’ experiences, turnaround times and adherence of non-genetic HCPs to the mainstream genetic testing protocol. We compared these outcomes to those of a control group receiving standard genetic care (pre-test counseling performed by a genetic counselor or clinical geneticist). So far, only four previous studies have evaluated genetic care of patients receiving mainstreamed genetic care in direct comparison to a valid control group, and for the majority with a limited number of patients in these groups [16, 18–20].

We showed that decisional conflict, anxiety, depression and distress were comparable for the patients in our intervention and control group. We did find differences in regret, discussed topics, and knowledge between the two groups. It is not surprising to find these differences between the two groups, as non-genetic HCPs did not have the same training as clinical geneticists. In addition, they have limited time during consultations to include pre-test genetic counseling. We think these differences are acceptable as long as patients do not experience high levels of decision regret or distress, and feel that they can make an informed choice whether or not to perform genetic testing.

The level of decision regret was significantly higher in our intervention group compared to our control group. Although no definite cut-off scores have been determined for decision regret so far, other studies have used a cut-off score of 25 to indicate strong levels of regret [33, 34]. In our study, the level of regret in both groups are far below this threshold (12.9 in the intervention group, 9.7 in the control group) and in line with the previous study of McLeavy et al. [17]. In addition, decision regret is measured on a scale of 0 to 100 and this three-point difference in level of regret seems clinically irrelevant.

The other psychosocial outcomes (decisional conflict, anxiety, depression and distress) were comparable between the two groups. Decisional conflict in both groups was far below the previously determined cut-off level of concern of 37.5 [35]. This is in line with the research of Richardson et al. [19]. In contrast, Yoon et al. did see a significantly higher decisional conflict in patients receiving pre-test counseling by a non-genetic HCP compared to patients receiving pre-test counseling by a genetic counselor or clinical geneticist [20]. However, in this study decisional conflict scores for both groups were also below the level of concern of 37.5, and therefore they concluded that this difference was clinically irrelevant. Anxiety and depression have not previously been evaluated in patients receiving mainstreamed genetic care. The levels of anxiety and depression we found in our study are comparable with the outcomes of Beek et al. [11]. They showed that patients who received pre-test counseling by a genetic counselor or clinical geneticist had a median anxiety level of 5.0 and a median depression level of 3.0 six months after diagnosis. Distress levels have been evaluated in a few studies and, as in our study, have been comparable between patients receiving mainstreamed genetic care and patients receiving pre-test counseling by a clinical geneticist or genetic counselor [16, 18–20].

For patients to make an informed decision, it is important that they are aware of the possible implications of a DNA test for themselves, but also for family members. Overall, knowledge about genetics was similar between the two groups, which is in line with previous studies [16, 19]. However, the statement that a sister with a pathogenic variant in an ovarian cancer gene has a 50% chance of having the same pathogenic variant was answered incorrectly by significantly more patients in our intervention group. However, for patients to make a well-informed decision whether or not to perform a DNA test, we believe it is sufficient to have general knowledge of possible implications for family members. Detailed information about inheritance patterns only becomes relevant when a pathogenic variant is identified, and for these patients post-test counseling is always performed by a genetics counselor or clinical geneticist.

Significantly fewer patients in the intervention group mentioned that the possible higher risk of breast cancer for patients with EOC carrying a pathogenic *BRCA1/2* variant was discussed during pre-test counseling. So far, only Colombo et al. also have assessed which topics were discussed during pre-test counseling, although they did not specifically ask about the possible higher risk of breast cancer [5]. We asked specifically about the risk of breast cancer for patients with ovarian cancer. Especially in patients suffering from advanced disease stage, potential breast cancer risk might not always be clinically relevant, and therefore not discussed during pre-test counseling. It is important for family members to be informed about the possible risk of breast cancer, but this is only relevant when a pathogenic variant is identified, for which all patients receive post-test counseling by a genetic counselor or clinical geneticist. When implementing a mainstream genetic testing pathway, we recommend educating non-genetic HCPs to include in their pre-test counseling the possible higher risk of breast cancer for patients with EOC carrying a pathogenic variant in a *BRCA* gene.

Overall, satisfaction with the genetic care pre- and post-test was high in both groups. We considered it foremost important to analyze patients who were unhappy with the care they received. Only four (4%) of the 105 patients felt that they had not had enough time to consider the advantages and disadvantages of a DNA test, which indicates that the majority (96%) of patients in our intervention group had enough time to consider the DNA test. Regarding the satisfaction with the way the test result was received, the majority of patients in the mainstream group (99%) considered it acceptable to receive this result in a letter. It is possible that patients in our intervention group would have chosen another way of receiving their test result if they had been offered a choice. However, providing post-test counseling to all patients via telephone or face-to-face consultation would be more time-consuming. Therefore, we foremost wanted to evaluate if receiving the test result in a letter was acceptable to patients.

The timing of genetic testing is important to consider, as patients might be eligible for primary treatment with PARP inhibitors if a pathogenic *BRCA* variant is identified [4, 36]. In this study, the mainstream genetic testing pathway resulted in a significant reduction in wait time to pre-test counseling, similar to other studies [6, 15, 19, 37]. This is beneficial for making early treatment decisions. However, only about 50% of patients in both groups in our study preferred to be offered genetic testing directly after being diagnosed with EOC. On the other hand, even though about 35% of patients might have preferred to be offered genetic testing in a later stage (e.g., after completion of treatment), they were still satisfied that they had been offered germline genetic testing. Timing of genetic testing should also be considered when implementing workflows that use tumor testing as a pre-test for germline genetic testing [38]. Given these differences in preferences regarding timing between our groups, it is important that non-genetic HCPs are aware of these differences and explore patients’ preferences during pre-test counseling. Any patient who expresses doubts about genetic testing during pre-test counseling should be referred to a genetics department for more extensive counseling in making a decision about whether or not to perform genetic testing.

It is important to identify those patients who might benefit from additional genetic testing or should be given advice about preventive measurements. We have shown that it is feasible for non-genetic HCPs to identify these patients by completing checklists, as these checklists were present in more than 95% of patient files. However, this system only works if patients are referred when indicated by the checklist, which was omitted for two patients in our study. For the implementation of future mainstream genetic testing initiatives, it is important to incorporate a procedure that ensures that all patients who require additional counseling are offered post-test counseling at a genetics department.

For the sustainability of a mainstream genetic testing pathway, it is important that it can be easily adapted to changes in gene panels. Indeed, our gene panel was expanded to include *BRIP1, RAD51C* and *RAD51D* and this could be easily adapted in the workflow [9]. Our training provided the basic tools to provide pre-test counseling that are also applicable to other genes.

The strengths of our study are the comparison of a mainstream genetic testing pathway with the standard genetic testing pathway from the patients’ perspective and the high participation rate in both groups (intervention group (79%) and control group (60%)). So far, most studies evaluating both mainstreamed and standard genetic care have evaluated only a small group of less than 50 patients receiving mainstreamed genetic care [16, 18, 19].

A limitation of our study is the design. Part of our control group was invited to participate retrospectively, which could be up to a year after pre-test counseling. Therefore, it is possible that there is some recall bias in our results. In addition, in the intervention group the mean age of diagnosis was higher, more patients were newly diagnosed at time of pre-test counseling and more patients had children. We expected the mean age and the number of newly diagnosed patients to be higher in this group because of an increased awareness of genetic testing amongst non-genetic HCPs. We cannot explain why more patients in the intervention group had children. We accounted for being newly diagnosed and having children as possible confounders by including these in our multivariate analyses. We did not ask patients about their family history, therefore we could not evaluate if this had any impact on our study outcomes. Another limitation is that we only evaluated patient experiences in our control group after receiving the test result. Therefore, we could not compare experiences between our intervention and control group after pre-test counseling.

Overall, this study demonstrates that the pre-test counseling provided in our mainstream genetic testing pathway enables patients to make a well-informed decision about genetic testing. Although we did find differences in genetic care between the two groups, patients receiving mainstreamed genetic care did not experience unacceptably high levels of distress or decision regret. In addition, all patients carrying a pathogenic variant or variant of unknown significance in our study were invited for post-test counseling at a genetics department. This ensured that all these patients received detailed information about the implications of their test result for themselves and their family members. We previously showed that, after completion of an online training module, non-genetic HCPs, such as gynecologic oncologists, feel motivated and competent to discuss and order germline genetic testing themselves [9]. This, in combination with the positive experiences of patients shown in this study, indicates that mainstream genetic testing provides a feasible and sustainable new care pathway for all patients with EOC. In training non-genetic HCPs, it is important to especially consider the possible higher risk of breast cancer for patients carrying a pathogenic variant in a *BRCA* gene. In addition, we recommend incorporating a procedure to ensure that all patients who require additional counseling are offered post-test counseling at a genetics department.

## Supplementary Information

**Additional file 1: Supplementary Table 1.** Knowledge of patients in the intervention and control group. For all statements patients could choose between ‘true’, ‘false’, and ‘don’t know’. *p ≤ 0.05.

## Data Availability

The datasets used and/or analyzed during the current study are available from the corresponding author on reasonable request.

## Abbreviations

EOC: Epithelial ovarian cancer
HCP: Healthcare professional
HADS: Hospital Anxiety and Depression Scale
DT: Distress Thermometer
MCG: Mainstreaming Cancer Genetics
