# Supplementary material for: Mainstream genetic testing for women with ovarian cancer provides a solid basis for patients to make a well-informed decision about genetic testing
Source: Hered Cancer Clin Pract. 2022 Sep 8;20:33. doi: 10.1186/s13053-022-00238-w (PMC9461259; doi:10.1186/s13053-022-00238-w)
Supplement: Supplementary file 1 — Additional file 1: Supplementary Table 1. Knowledge of patients in the intervention and control group. For all statements patients could choose between ‘true’, ‘false’, and ‘don’t know’. *p ≤ 0.05. [file 13053_2022_238_MOESM1_ESM.docx]

**Supplementary Table 1. Knowledge of patients in the intervention and control group**

| Questions | Options | Intervention group, T1  n = 96 | Control group  n = 91 | p-value |
| --- | --- | --- | --- | --- |
| All women with a pathogenic variant (gene alteration) in an ovarian cancer gene will someday develop ovarian cancer, n (%) | - correct - incorrect - missing | 49 (51.0)  46 (47.9)  1 (1.0) | 41 (45.1)  50 (54.9)  0 | 0.374 |
| A woman without a pathogenic variant (gene alteration) in an ovarian cancer gene can still develop ovarian cancer, n (%) | - correct - incorrect - missing | 75 (78.1)  20 (20.8)  1 (1.0) | 72 (79.1)  19 (20.9)  0 | 0.977 |
| A woman with a pathogenic variant (gene alteration) in an ovarian cancer gene can pass this alteration on to her children, n (%) | - correct - incorrect - missing | 79 (82.3)  17 (17.7)  0 | 80 (87.9)  11 (12.1)  0 | 0.284 |
| A woman may have inherited a pathogenic variant (gene alteration) in an ovarian cancer gene from her father, n (%) | - correct - incorrect - missing | 45 (46.9)  51 (53.1)  0 | 45 (49.5)  46 (50.5)  0 | 0.725 |
| A woman who has a sister with a pathogenic variant (gene alteration) in an ovarian cancer gene has a 50% chance (1 in 2) of having this gene alteration as well, n (%) | - correct - incorrect - missing | 36 (37.5)  58 (60.4)  2 (2.1) | 54 (59.3)  33 (36.3)  4 (4.4) | 0.002* |

*For all statements patients could choose between ‘true’, ‘false’, and ‘don’t know’.*

*T1: questionnaire sent to patients approximately four weeks after receiving their DNA test result.*

**p ≤ 0.05*
